# Immune Activation and Bacterial Translocation: A Link between Impaired Immune Recovery and Frequent Visceral Leishmaniasis Relapses in HIV-Infected Patients

**DOI:** 10.1371/journal.pone.0167512

**Published:** 2016-12-01

**Authors:** Maria Luciana Silva-Freitas, Glaucia Fernandes Cota, Talia S. Machado-de-Assis, Carmem Giacoia-Gripp, Ana Rabello, Alda M. Da-Cruz, Joanna R. Santos-Oliveira

**Affiliations:** 1 Laboratório Interdisciplinar de Pesquisas Médicas – Instituto Oswaldo Cruz – FIOCRUZ, Rio de Janeiro, Rio de Janeiro, Brazil; 2 Laboratório de Pesquisas Clinicas e Políticas Públicas em Doenças Infecciosas e Parasitárias – Centro de Pesquisas René Rachou – FIOCRUZ, Belo Horizonte, Minas Gerais, Brazil; 3 Hospital Eduardo de Menezes – Fundação Hospitalar do Estado de Minas Gerais-FHEMIG, Belo Horizonte, Minas Gerais, Brazil; 4 Laboratório de AIDS e Imunologia – Instituto Oswaldo Cruz – FIOCRUZ, Rio de Janeiro, Rio de Janeiro, Brazil; 5 Núcleo de Ciências Biomédicas Aplicadas, Instituto Federal de Educação, Ciência e Tecnologia – IFRJ, Rio de Janeiro, Rio de Janeiro, Brazil; Taibah University, SAUDI ARABIA

## Abstract

The maintenance of chronic immune activation due to leishmaniasis or even due to microbial translocation is associated with immunosenescence and may contribute to frequent relapses. Our aim was to investigate whether patients with HIV-associated visceral leishmaniasis (VL/HIV) who experience a single episode of VL have different immunological behaviors in comparison to those who experience frequent relapses. VL/HIV patients were allocated to non-relapsing (NR, n = 6) and relapsing (R, n = 11) groups and were followed from the active phase of VL up to 12 months post-treatment (mpt). The patients were receiving highly active antiretroviral therapy (HAART) and secondary prophylaxis after VL therapy. During active VL, the two groups were similar in all immunological parameters, including the parasite load. At 6 and 12 mpt, the NR group showed a significant gain of CD4^+^ T cells, a reduction of lymphocyte activation, and lower soluble CD14 and anti-*Leishmania* IgG3 levels compared to the R group. The viral load remained low, without correlation with the activation. The two groups showed elevated but similar percentages of senescent T cells. These findings suggest a decreased ability of the R group to downmodulate immune activation compared to the NR group. Such functional impairment of the effector response may be a useful indicator for predicting clinical prognosis and recommending starting or stopping secondary prophylaxis.

## Introduction

The increasing frequency of HIV-associated visceral leishmaniasis (VL/HIV) has become a significant problem in East Africa, Brazil and India. Brazil presents the highest number of co-infection cases in South America, with 8.5% of the HIV-infected individuals in the country being co-infected with VL in 2012 [1]. Both diseases profoundly impair the immune mechanisms involved in the control of infections, which makes the outcomes of VL/HIV very poor. Compared to VL patients without HIV/AIDS, VL/HIV-co-infected patients show a less robust, slower clinical response to treatment and higher frequencies of drug toxicity, relapses and mortality [1–4].

The overall immunological reconstitution observed after highly active antiretroviral therapy (HAART) introduction, at least in countries where it is available, has reduced the incidence of opportunistic infections, including the incidence of *Leishmania*/HIV co-infection [5–8]. In addition to decreased virus-mediated immune activation, a marked increase in the CD4^+^ T cell counts probably also strengthens the effector mechanisms associated with parasite control [8,9]. Moreover, there is *in vitro* evidence that antiretrovirals, and particularly protease inhibitors (PIs), have an inhibitory effect on the evolutionary forms of *Leishmania (L*.*) infantum* [10,11]. This finding indicates another factor that could contribute to the decline of new VL cases among HIV-infected people receiving HAART [10–12].

Despite reducing new VL cases among HIV/AIDS patients, HAART does not prevent relapses [2,13,14], which still pose a challenge to clinical management. Parasite reactivation occurs even in patients with a good response to HAART, i.e., with an undetectable viral load and an increased CD4^+^ T cell count [15,16]. Early studies soon after the HAART era [17] did not show significant differences when these virological and immunological parameters were compared between relapsing and non-relapsing patients receiving HAART. This fact suggests that HAART does not prevent recurrences, especially in those individuals with previous VL episodes.

Co-infected patients receiving HAART, even after anti-*Leishmania* treatment, maintain a low CD4^+^ T cell count and higher levels of cellular activation despite viral suppression [16,18]. Considering that such pathogenic features are similar between VL and HIV, these results reinforce the concept that VL may contribute to worsening the immunosuppression induced by HIV infection, accelerating progression to AIDS. In addition, the quality of the *Leishmania*-specific immune response may remain impaired in *Leishmania*/HIV patients receiving HAART due to reduced T cell proliferative capacity and deficient interferon (IFN)-γ production [19,20]. Consequently, this deficient parasite control may favor the spread of *L*. *infantum* to unexpected sites and the appearance of atypical clinical features. In this scenario, amastigotes have been recovered from skin [20,21] and from gut-associated lymphoid tissue (GALT) [22,23].

There is strong evidence that chronic cellular activation is a crucial immunopathogenic mechanism not only in HIV infection but also in VL [24–27]. As a result, VL/HIV-co-infected patients present increased levels of activated CD38^+^CD8^+^ T lymphocytes [18] as well as elevated levels of pro-inflammatory cytokines [28,29]. Unexpectedly, this heightened cellular activation remains despite successful control of the viral and parasite loads [28] by HAART and anti-*Leishmania* therapy, respectively. Microbial translocation and even parasite persistence in the bone marrow or lymphoid organs have been identified as possible factors related to the maintenance of immune activation [28,30]. In this context, there is strong evidence that secondary anti-*Leishmania* prophylaxis helps to maintain the clinical remission of VL in HIV-infected patients [2,3,10,14]. We hypothesize that controlling the parasite load could contribute to the reduction of cellular activation during *Leishmania*/HIV co-infection. In turn, this control could strengthen the effector immune response, diminishing the occurrence of relapses.

Another consequence of immune activation is accelerated and premature aging of the immune system [31–33], even in HIV-infected patients with viral load suppression [33]. This premature aging is characterized by an exhaustion of primary immune resources, decreased thymic output and an accumulation of terminally differentiated cells, similar to what occurs in healthy elderly subjects [34,35]. Thus, co-infected patients may experience acceleration of the degree of immunosenescence, providing an additional factor that decreases the effector immune response to *Leishmania* antigens and contributes to frequent relapses.

Previous results from our group have shown that VL/HIV/AIDS patients display high levels of cellular activation during both clinical remission of leishmaniasis and HAART [18]. However, it is crucial to determine whether and how secondary anti-*Leishmania* prophylaxis in patients receiving HAART can favor T cell immune reconstitution and reduce activation levels, which together can help to prevent further relapses.

The aim of this study was thus to determine the T cell activation and senescence profiles presented by VL/HIV-co-infected patients during 12 months of prospective follow-up while under viral and parasitological therapy in order to identify immunological parameters that can be used to predict VL relapse.

## Methods

### Study design and participants

Eighteen *Leishmania*/HIV-co-infected patients were recruited for a prospective cohort study carried out in Belo Horizonte, Minas Gerais, Brazil, from February 2011 to March 2013. The VL/HIV-co-infected patients were separated into two groups: those who had experienced only one VL episode throughout life (non-relapsing (NR) group) and those experiencing more than one VL episode, either previously or during the prospective follow-up (relapsing (R) group). HIV-infected patients without a history of VL (n = 16) and healthy subjects without either infection (n = 12) were also included as controls.

The diagnosis of VL was confirmed by a parasitological exam of bone marrow aspirates from all patients. Clinical evaluation and an immune response panel test were performed before the VL treatment and every two months during treatment for one year. Approval for this study was obtained from the Ethical Review Board of Eduardo de Menezes Hospital—Hospital Foundation of the State of Minas Gerais and from Centro de Pesquisas René Rachou—Fundação Oswaldo Cruz. Patients with clinical symptoms and parasitological confirmation of active VL were included only after appropriate written informed consent was obtained. The first-line treatment for VL in the HIV-infected patients was intravenous amphotericin B deoxycholate for 4 weeks. After VL treatment, for all patients with CD4^+^ T cell counts less than 350 cells/mm^3^, secondary prophylaxis with amphotericin B was offered every two weeks.

### Immunological and virological assessments

Absolute T lymphocyte counts were determined using the BD Multitest monoclonal antibodies anti-CD45-PerCP, anti-CD3-FITC, anti-CD4-APC, and anti-CD8-PE (BD Biosciences, Franklin Lakes, NJ, USA) according to the manufacturer’s instructions and as described previously by Santos-Oliveira et al. (2013) [28]. The counts were acquired using a FACSCalibur and were analyzed with Multiset software (BD^®^). The results are expressed as the number of cells per cubic millimeter (cells/mm^3^). The plasma HIV RNA levels were measured using real-time quantitative PCR (RT-PCR) (Abbott^®^, Des Plaines, IL, USA) according to the manufacturer’s recommendations. The detection range was from 40 to 10,000,000 RNA copies/mL plasma. Assay results below 40 copies/mL are expressed as undetectable or below the detection limit.

### *Leishmania* parasite load assessment

Total DNA was extracted from the peripheral blood of patients using the QIAamp DNA Blood Mini Kit (Qiagen^®^ GMbH, Hilden, Germany). Two independent assays for the detection and quantification of *Leishmania* spp. and human DNA were performed using the StepOnePlus^™^ Real-Time PCR System (Life Technologies^®^, Carlsbad, CA, USA) as described in a previous study [4]. For the *Leishmania* assay, TaqMan real-time PCR was performed using the small-subunit ribosomal RNA (SSU rRNA) gene as the target DNA, as described by Wortmann et al. (2002) [36], according to the protocol described by Gomes and others (2012) [37]. Standard curves were prepared for each assay using known quantities of pCR-4 TOPO vector (Life Technologies^®^) containing the cloned 120-bp human gene *ACTB* and the 67-bp *L*. *infantum* SSU rRNA fragment. The parasite load is expressed as the *Leishmania* DNA load (the relative copy number of the SSU rRNA fragment) normalized to the reference gene *ACTB*, as reported by Overbergh and others (1999) [38]. The *ACTB* copy numbers for the target samples were divided by the highest *ACTB* value obtained in the experiment to generate the correction factor used for the normalization.

### Isolation of peripheral blood mononuclear cells (PBMCs) and cytofluorometric assays

PBMCs were obtained as described elsewhere [18] and were used for cytofluorometric assays. The PBMCs were labeled with the following human monoclonal antibodies: anti-CD3-APC, anti-CD4-PerCP or anti-CD4-FITC and anti-CD8-APC or anti-CD8-FITC, anti-CD38-PE and anti-HLA-DR-PerCP, and anti-CD57-FITC and anti-CD27-PE. All antibodies were purchased from BD^®^ Biosciences. At least 20,000 events in the lymphocyte gate were acquired on a FACSCalibur and were analyzed with CellQuest^™^ software (BD^®^ Biosciences). The analysis region was first established by gating on the CD3^+^ T lymphocytes based on a forward scatter *versus* fluorescence dot plot. After that, the results were determined as the percentages of both activated T cells within the CD3^+^ T lymphocyte population (CD38^+^HLA-DR^+^CD4^+^ and CD38^+^HLA-DR^+^CD8^+^ T cells) (S1 Fig) and senescent T cells (CD57^+^CD27^-^CD4^+^ and CD57^+^CD27^-^CD8^+^ T cells) (S2 Fig).

### Quantification of lipopolysaccharide (LPS) and soluble CD14 (sCD14)

The samples were diluted in endotoxin-free water, and LPS levels were quantified using a commercial assay kit (Limulus Amebocyte Lysate QCL-1000; Cambrex^®^, Milan, Italy). The results are expressed as picograms per milliliter (pg/mL), and the sensitivity level was 10 pg/mL. sCD14 levels were quantified by ELISA using the Human sCD14 Quantikine ELISA Kit (R&D^®^ Systems, Minneapolis, MD, USA). The results are expressed as nanograms per milliliter (ng/mL), and the minimum detection limit was 125 pg/mL.

### Anti-*Leishmania* immunoglobulin detection

An ELISA was performed as previously described by Fagundes-Silva et al. (2012), with certain modification [39]. Briefly, the only difference was the antigen, since *L*. *(L*.*) infantum* (MHOM/BR/1974/PP75) soluble promastigote (40 μg/mL) was used to coat a polystyrene flat-bottom microtiter plate (Nunc-Immuno, Roskilde, Denmark). In this assay, diluted peroxidase-conjugated mouse monoclonal anti-human immunoglobulin G (IgG) (1:1000) (Invitrogen, San Francisco, CA, USA) and diluted monoclonal anti-human IgG1 (1:200) and IgG3 (1:400) (Zymed Laboratories Inc., San Francisco, CA, USA) were used. The absorbance was measured with a Benchmark microplate reader (Bio-Rad Laboratories, Hercules, CA, USA) at 492 nm and is expressed as an ELISA index (EI).

### Statistical analyses

Continuous variables are expressed as medians and interquartile ranges (IQRs). Comparisons were performed using unpaired Student’s t-tests for normally distributed variables and Wilcoxon tests for paired variables with skewed distributions. Spearman’s test was used for correlation analysis, and a chi-square test was used to compare categorical variables. The statistical analyses were performed using SPSS^®^ version 16 and GraphPad Prism software (version 5.0, San Diego, CA, USA). A paired test (Kruskal-Wallis test) was used because the same patients are observed when evaluating data prospectively. Differences were considered statistically significant when the p value was <0.05.

## Results

### Clinical characteristics and evolutionary course of VL/HIV patients

The demographic characteristics and clinical evolution of all patients studied are shown in Table 1. The mean patient age was 37.5±0.7 years. Fourteen patients were men (78%), and half of all patients (nine patients) had already experienced a prior VL episode. In 15 patients, HIV infection had been diagnosed before the diagnosis of primary VL, and most of the patients (14 patients) had already been treated with HAART. However, out of ten patients using HAART for more than 3 months, only 7 were taking their medications on a regular basis.

**Table 1:** Clinical evolution and demographic characteristics of visceral leishmaniasis/HIV (VL/HIV)-co-infected patients.

Patients’ initials	Age, sex	Time since HIV infection diagnosis (months)	HAART use before first VL episode	Time between HIV and VL diagnoses (months)	Time since the first VL infection diagnosis (months)	Previous VL (number of episodes)	VL treatment	Secondary prophylaxis	Total follow-up time in the study	Clinical follow-up
**GBS (HLV01)**	53 years, male	4	No	4	NA	No	Amphotericin B deoxycholate for 20 days	Amphotericin B deoxycholate biweekly for two months (until CD4 count recovery)	12 months	No VL relapse
**WLA (HLV03)**	38 years, male	179	Yes	179	NA	No	Amphotericin B deoxycholate for 3 days, followed by liposomal amphotericin 20 mg/kg total dose	Patient abandoned follow-up and received no prophylaxis	6 months (death)	VL relapse at 6-month follow-up and death
**JRO (HLV05)**	25 years, female	78	No	49	29	Yes (1)	Liposomal amphotericin 20 mg/kg total dose	Irregular use of prophylaxis	12 months	No VL relapse
**MF (HLV06)**	51 years, male	4	No	4	NA	No	Amphotericin B deoxycholate for 20 days	Amphotericin B deoxycholate biweekly for 20 months (patient requested suspension of prophylaxis when CD4 count stabilized at approximately 200 cells/mm3)	12 months	No VL relapse
**APS (HLV07)**	33 years, female	20	Yes	20	NA	No	Amphotericin B deoxycholate for 20 days	Amphotericin B deoxycholate biweekly for 12 months (patient requested suspension of prophylaxis when CD4 count stabilized at approximately 250 cells/mm3)	12 months	No VL relapse
**RAS (HLV09)**	39 years, male	41	Yes	21	19	Yes (1)	Liposomal amphotericin 20 mg/kg total dose	Liposomal amphotericin biweekly on regular basis	12 months	VL relapse at 4- and 12-month follow-up
**LPO (HLV010)**	35 years, male	27	Yes	17	9	Yes (1)	Liposomal amphotericin 20 mg/kg total dose	Amphotericin B deoxycholate biweekly for 7 months (until CD4 count recovery)	12 months	No VL relapse
**CMS (HLV012)**	40 years, male	134	No	52	82	Yes (4)	Liposomal amphotericin 20 mg/kg total dose	Liposomal amphotericin biweekly on regular basis	6 months (death)	VL relapse at 6-month follow-up and death
**CMRR (HLV013)**	37 years, female	5	No	5	NA	No	Amphotericin B deoxycholate for 5 days, followed by liposomal amphotericin 20 mg/kg total dose	Amphotericin B deoxycholate biweekly for 10 months (patient requested suspension of prophylaxis when CD4 count stabilized at approximately 200 cells/mm3)	12 months	No VL relapse
**PJS (HLV016)**	41 years, male	31	Yes	31	NA	No	Amphotericin B deoxycholate for 20 days	Amphotericin B deoxycholate biweekly for 4 months (until CD4 count recovery)	12 months	No VL relapse
**WMC (HLV017)**	38 years, male	14	Yes	14	NA	No	Amphotericin B deoxycholate for 20 days	Irregular use of prophylaxis (amphotericin B deoxycholate biweekly), with use for 18 months (until CD4 count recovery)	12 months	VL relapse at 4-, 6- and 12-month follow-up
**PRP (HLV019)**	45 years, male	69	Yes	69	NA	No	Amphotericin B deoxycholate for 20 days	Irregular use of prophylaxis (amphotericin B deoxycholate biweekly), with use for 13 months (until CD4 count recovery)	12 months	VL relapse at 8-month follow-up
**JTS (HLV021)**	45 years, male	139	Yes	113	25	Yes (4)	Liposomal amphotericin 20 mg/kg total dose	Irregular use of prophylaxis (amphotericin B deoxycholate biweekly)	12 months	VL relapse at 2-, 8- and 12-month follow-up
**VPGS (HLV022)**	21 years, male	25	No	1	24	Yes (1)	Liposomal amphotericin 20 mg/kg total dose	Amphotericin B deoxycholate biweekly for 13 months (until CD4 count recovery)	12 months	VL relapse at 6-month follow-up
**AMP (HLV023)**	39 years, male	239	Yes	117	121	Yes (7)	Liposomal amphotericin 20 mg/kg total dose	Liposomal amphotericin weekly on regular basis	12 months	VL relapse at 6- and 12-month follow-up
**AMGN (HLV024)**	37 years, female	155	Yes	107	47	Yes (1)	Amphotericin B deoxycholate for 20 days	Irregular use of amphotericin B deoxycholate biweekly for 4 months	4 months (lost to follow-up)	No VL relapse during short follow-up
**DCLS (HLV025)**	30 years, male	2	No	2	NA	No	Amphotericin B deoxycholate for 25 days	Amphotericin B deoxycholate biweekly for 13 months (until CD4 count recovery)	12 months	No VL relapse
**ACL (HLV026)**	52 years, male	128	No	0	128	Yes (2)	Liposomal amphotericin 20 mg/kg total dose	Amphotericin B deoxycholate biweekly for 8 months (until CD4 count recovery)	12 months	No VL relapse

NA: not applicable

Patients with more than one VL episode (R group, n = 12) had lower normalization rates for clinical and laboratory parameters at 3 months after treatment (p = 0.02), a lower *Leishmania* clearance rate after anti-*Leishmania* treatment (p = 0.01) and a lower CD4^+^ T lymphocyte count at the 12-month follow-up in comparison to those who had experienced only one VL episode (NR group, n = 6). In contrast, no difference was observed between the R and NR groups in terms of the proportion of patients who had used protease inhibitors or who had presented other opportunistic infections in the past.

Additionally, R patients had a different immunological profile in terms of cellular activation compared to NR patients at the 12-month follow-up. Interestingly, possibly related to these differences, we observed a longer time between the HIV and VL diagnoses in the R group than in the NR group (median: 51 *versus* 5 months, p = 0.05). Similarly, HIV infection had been diagnosed longer before VL infection in the R group than in the NR group (median: 103 (95%CI 29.5–141) *versus* 5 (95% CI 3.5–21.25) months, p = 0.007) (Table 1).

### The occurrence of several episodes of VL impairs the degree of immune reconstitution in VL/HIV patients, independent of the virological and parasitological loads

**Fig 1:**
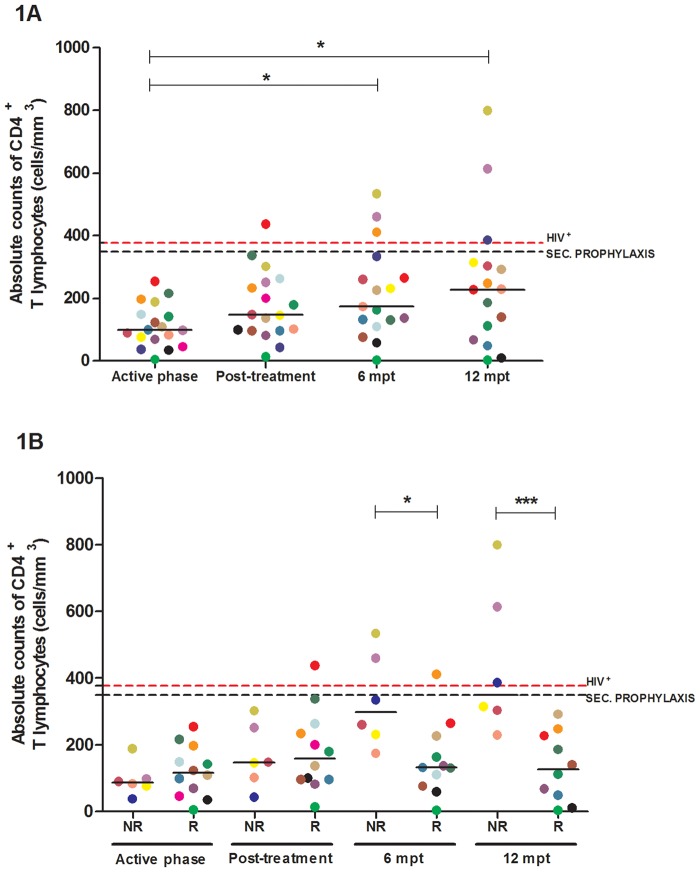
Immune constitution of visceral leishmaniasis/HIV (VL/HIV)-co-infected patients. Absolute counts of CD4^+^ T lymphocytes during the prospective follow-up of co-infected patients (A) and after patient allocation into a non-relapsing (NR) group, encompassing those with a single episode of VL, and a relapsing (R) group, encompassing those with disease relapse during the follow-up or even before being enrolled in the study (B). The black dashed line represents the recommended limit for the establishment of secondary prophylaxis (350 cells/mm^3^). The red dashed line is the median value of the CD4^+^ T cell counts of the HIV-positive controls (377 cells/mm^3^). Each symbol represents one patient, and the color refers to the same patient at different stages of follow-up. The horizontal bars represent the median values. 6 mpt: six months post-treatment; 12 mpt: 12 months post-treatment. Asterisks denote a statistically significant difference between the phases of follow-up or between the NR and R groups, *p<0.05; ***p<0.001.

All co-infected patients had low CD4^+^ T cell numbers during the active phase (median: 98 cells/mm^3^; IQR: 63–159 cells/mm^3^) compared to HIV-mono-infected patients (median: 377 cells/mm^3^; IQR: 222–450 cells/mm^3^) (Fig 1A). In a prospective evaluation, most of the patients continued to have counts less than 350 CD4^+^ T cells/mm^3^, which was the criterion used for the introduction and maintenance of secondary prophylaxis in this study. However, the VL/HIV-NR group had a significant gain of CD4^+^ T cells at the prospective follow-up six months post-treatment (mpt) (*p<0.05) compared to the VL/HIV-R group (Fig 1B), who maintained the same values as those observed in the active phase of the disease (Fig 1B).

The HIV viral load did not seem to be related to CD4^+^ T cell depletion because it was low or undetectable in 12 of 18 co-infected patients already in the active phase of VL, regardless of whether they belonged to the NR or R group (S1 Table). Similarly, recurrences occurred independently of the viral load because certain patients maintained undetectable HIV RNA copy numbers at the moment of relapse as well as at the end of the 12-month follow-up period (n = 4, S1 Table). Moreover, VL/HIV-NR patients had undetectable parasite loads soon after anti-*Leishmania* treatment. In all of these patients except for one, the undetectable levels were maintained up to the 12-month follow-up. In relation to VL/HIV-R patients, 5 of the 9 evaluated patients still had detectable parasite burdens in the periods 12 months subsequent to treatment (S1 Table), suggesting that parasite persistence at the end of treatment may indicate an increased risk of future relapses.

### The degree of immune activation in VL/HIV patients seems to be related to the occurrence of VL episodes

In a previous transversal study of VL/HIV patients [18,28], a multivariate analysis indicated that “leishmaniasis disease,” but not parasite load, and microbial translocation were the two main risk factors associated with the activation status.

**Fig 2:**
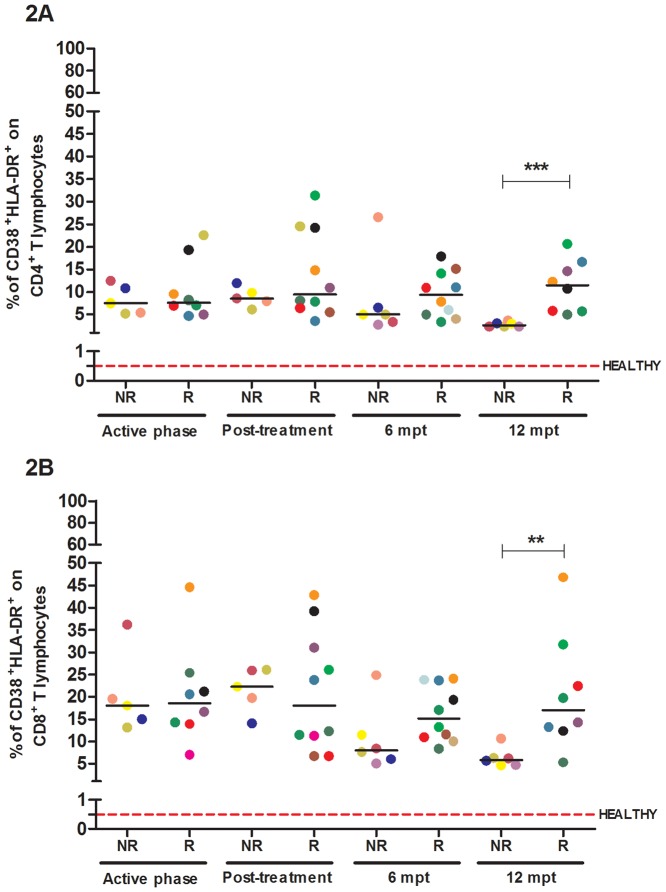
Activation levels of T lymphocyte subpopulations in visceral leishmaniasis/HIV (VL/HIV)-co-infected patients. Percentages of activated CD4^+^ (A) and CD8^+^ (B) T lymphocytes in relapsing (R) and non-relapsing (NR) co-infected patients. The red dashed line represents the median levels of activated CD4^+^ T and CD8^+^ T cells in healthy controls (median: 0.5%, interquartile ranges: 0.15–1.77% and 0.23–5.92%, respectively). Each symbol represents one patient, and the color refers to the same patient at different stages of follow-up. The horizontal bars represent the median values. 6 mpt: 6 months post-treatment; 12 mpt: 12 months post-treatment. Asterisks denote a statistically significant difference between the NR and R groups, **p<0.01; ***p<0.001.

In the present study, the percentages of activated T lymphocytes were comparable between the VL/HIV-NR and VL/HIV-R groups in the early stages of the follow-up. As expected, CD8^+^ T lymphocytes (Fig 2B) displayed higher activation levels than CD4^+^ T cells (Fig 2A). The CD8^+^ T and CD4^+^ T cells of VL/HIV-NR patients had lower levels of activation by the end of the clinical follow-up period (p<0.05). At the sixth month, the VL/HIV-NR group showed a trend towards decreased activation levels, but a significant reduction was observed at 12 mpt in this group compared to the VL/HIV-R group (**p<0.01).

**Fig 3:**
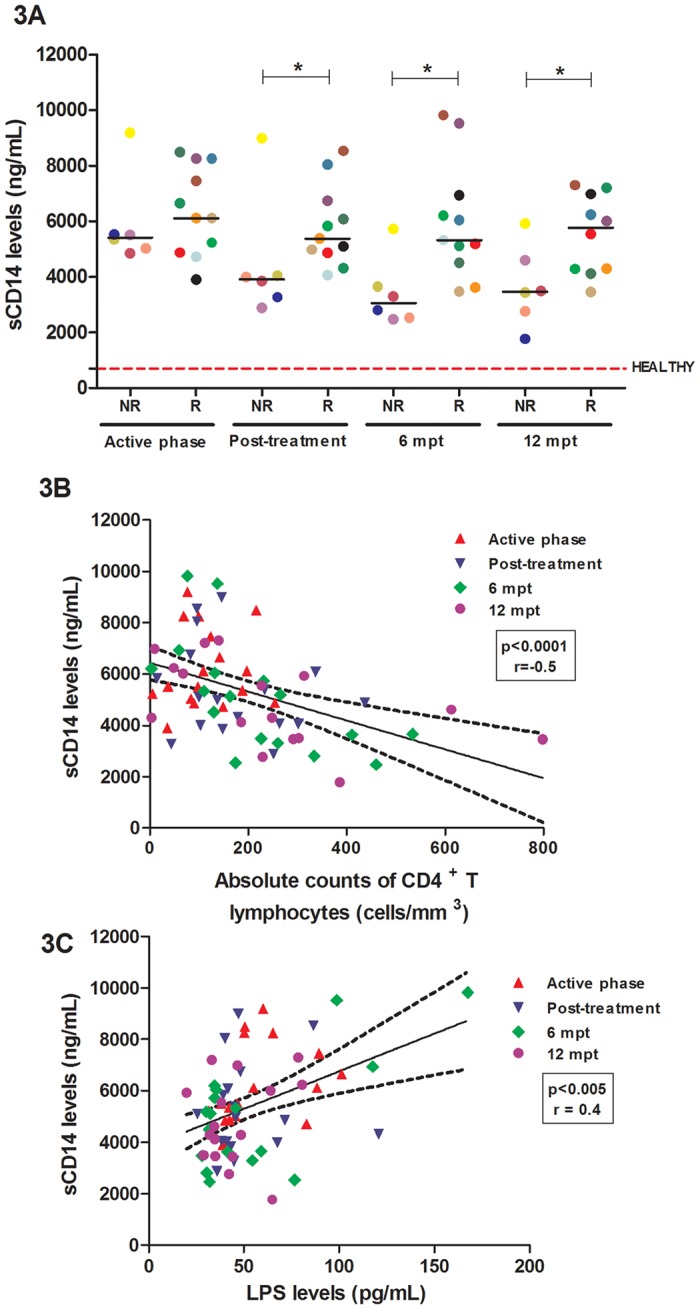
Relationship between plasma factors related to microbial translocation and the immune status of visceral leishmaniasis/HIV (VL/HIV)-co-infected patients. Plasma soluble CD14 (sCD14) level assessment in the relapsing (R) and non-relapsing (NR) groups during all the follow-up visits (A). Negative correlation between sCD14 levels and the absolute counts of CD4^+^ T lymphocytes (B) in co-infected patients (Spearman correlation, p<0.0005; r = -0.5). Positive correlation between sCD14 levels and LPS levels (C) in co-infected patients (Spearman correlation, p<0.001; r = 0.4). The red dashed line represents the median value of the sCD14 levels (median: 699 ng/mL; interquartile range: 155–1525 ng/mL). Each symbol represents one patient, and the color refers to the same patient at different stages of follow-up. The horizontal bars represent the median values. 6 mpt: 6 months post-treatment; 12 mpt: 12 months post-treatment. Asterisks denote a statistically significant difference between the NR and R groups, *p<0.05.

Furthermore, the R group had higher sCD14 levels compared to the NR group during all phases of follow-up, and this difference was already significant immediately post-treatment (*p<0.05) (Fig 3A). As expected, the sCD14 levels were positively correlated with the LPS levels in all phases of evaluation (p<0.001; r = 0.42) (Fig 3C). Additionally, a significant negative correlation was observed for the absolute CD4^+^ T cell counts (Fig 3B). A high degree of innate activation is also related to immune impairment because the release of sCD14 is dependent on LPS-mediated macrophage activation.

### Reduction of anti-*Leishmania* IgG3 levels is a predictor of the maintenance of clinical remission in VL/HIV patients

**Fig 4:**
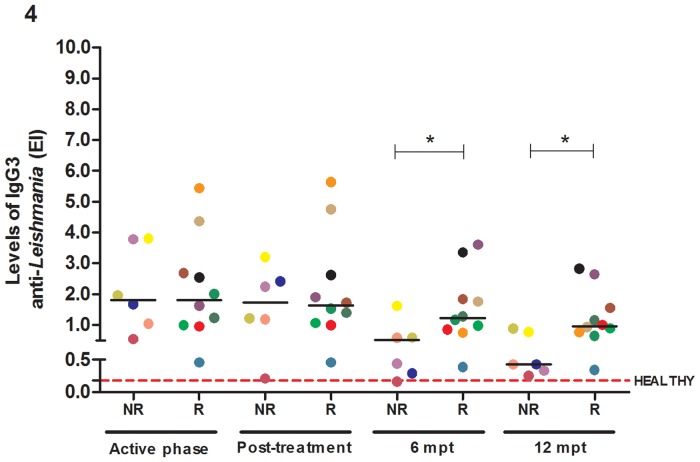
Titers of the anti-*Leishmania infantum* immunoglobulin G3 (IgG3) isotype in visceral leishmaniasis/HIV (VL/HIV)-co-infected patients. IgG3 levels in the relapsing (R) and non-relapsing (NR) groups during the entire follow-up. The red dashed line represents the median value of the IgG3 levels in healthy controls (median: 0.18; interquartile range: 0.1–0.3). Each symbol represents one patient, and the color refers to the same patient at different stages of follow-up. The horizontal bars represent the median values. 6 mpt: 6 months post-treatment; 12 mpt: 12 months post-treatment. Asterisks denote a statistically significant difference between the NR and R groups, *p<0.05.

Similarly, to how the T cell activation levels and macrophage activation status were evaluated, the latter based on sCD14 levels, B lymphocyte activation was evaluated by quantitation of the levels of anti-*Leishmania* IgG and its subclasses (IgG1 and IgG3). The VL/HIV-R group presented high levels of anti-*Leishmania* IgG and IgG1 compared to the VL/HIV-NR group throughout the study (S3 Fig). In contrast, the anti-*Leishmania* IgG3 levels were similarly elevated in the two groups in the early stages of follow-up but significantly decreased in the NR group in comparison to the R group at six and 12 mpt (*p<0.05) (Fig 4).

### Immunosenescence levels in VL/HIV patients are elevated independently of frequent episodes of VL relapses

**Fig 5:**
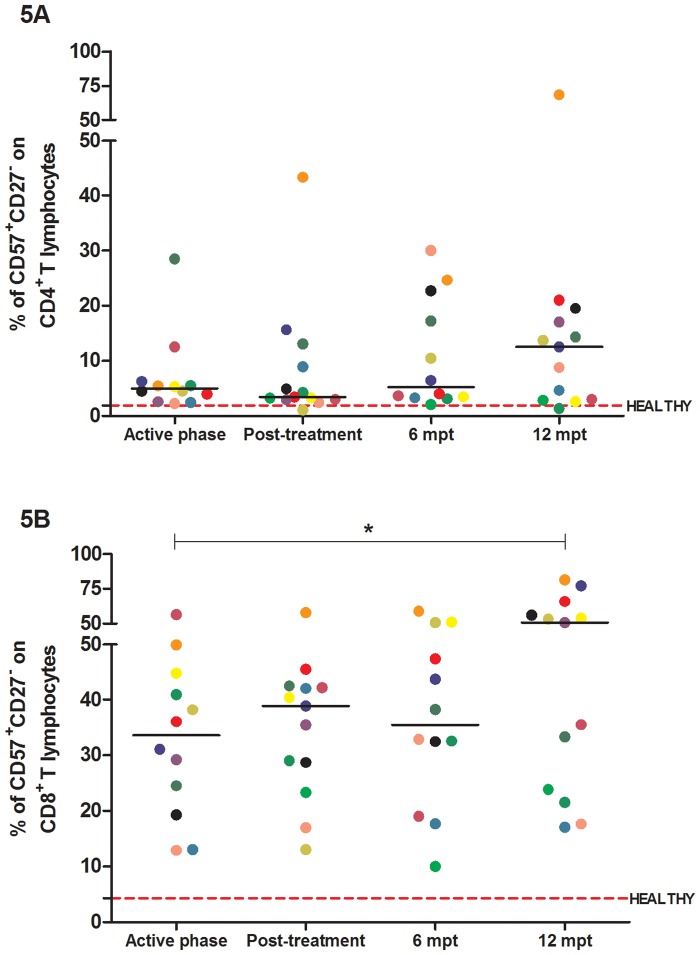
Immunosenescence levels in visceral leishmaniasis/HIV (VL/HIV)-co-infected patients. Percentages of senescent CD4^+^ (A) and CD8^+^ (B) T cells in co-infected patients during the prospective follow-up of co-infected patients. The red dashed line represents the median value of the percentage of senescent CD8^+^ T cells in healthy controls (medians: 1.8% and 4.2%; interquartile ranges: 0.3–4.0% and 2.6–10.1%, respectively). Each symbol represents one patient, and the color refers to the same patient at different stages of follow up. The horizontal bars represent the median values. 6 mpt: 6 months post-treatment; 12 mpt: 12 months post-treatment.

The CD57^+^CD27^-^ phenotype is found among senescent CD8^+^ and CD4^+^ T cells. In general, the co-infected patients presented higher percentages of senescent CD8^+^ T lymphocytes during all phases of follow-up. These values were higher than those observed in healthy controls of the same age (median: 4.2%; IQR: 2.6–10.1%), with a trend towards an increase by the end of follow-up (Fig 5B). Despite the lower percentages, the same result was observed in the subpopulation of senescent CD4^+^ T lymphocytes (Fig 5A). However, the percentages of senescent T lymphocytes were elevated in both the VL/HIV-NR and the VL/HIV-R groups, without any difference between them [data not shown].

These results suggest that VL/HIV patients who had experienced multiple VL relapses previously or during the clinical follow-up showed different immunological parameters compared to those who had experienced a single episode of active disease, even though both groups had controlled viral loads and received secondary prophylaxis.

## Discussion

Earlier cross-sectional studies identified *Leishmania* infection as a cofactor for heightening the activation status in HIV patients, independent of the viral or parasite load [18,28]. The present study confirmed that co-infected patients with active VL (NR or R group) have low CD4^+^ T cell counts, high levels of cellular activation and microbial translocation and elevated parasitemia. In contrast to a previous study, which evaluated a single parameter [30], herein, the patient’s prior history of VL, the current immunological state of the patient at each visit and the final outcome of therapy during secondary prophylaxis were all taken into account.

Recognition of the rationale underlying the frequent episodes of VL in the R patients is fundamental to designing preventive strategies. Our group has previously reported that diminished levels of cellular activation were stably maintained in a co-infected patient during the remission phase over a 12-month prospective follow-up period [20]. However, reactivation episodes were again marked by increased activation along with progressively lower levels of specific IFN-*γ* in response to parasite antigens [20]. Herein, a positive correlation between the number of relapses and the degree of cellular activation was observed in co-infected patients (p<0.05 and r = 0.80, data not shown). These results suggest that repeated VL relapses may worsen the effector immune response and consequently its ability to control parasites, resulting in a vicious cycle. These data reinforce the importance of epidemiological surveillance as well as the early diagnosis and treatment of VL.

The similarity in the impairment of immunological parameters between the NR and R groups at the beginning of follow-up indicates that the parasite antigens released during active disease were equally present in these groups, despite different clinical outcomes. In addition, longer exposure to HIV infection and a longer period between HIV diagnosis and the first active VL episode, as observed in the R group in comparison to the NR group, seem to be crucial factors that predispose patients to VL recurrence. The longer exposure could be related to the maintenance of high levels of activation, with consequent ongoing immune suppression and recurrence of disease.

Herein, the NR group displayed a significant decrease in the degree of activation at 6 and 12 mpt. This change could not have been due only to the control of *Leishmania* infection because the R group also had a reduced parasite load after anti-*Leishmania* treatment and secondary prophylaxis over 12 months. Despite this reduction, only four of the 11 co-infected patients in the R group still had low but detectable *Leishmania* kDNA copy numbers in the peripheral blood at the end of follow-up. This observation reinforces the concept that a continuous low level of *Leishmania* parasitemia can occur in treated patients despite an adequate clinical response to specific therapy. This phenomenon can lead to a clinical condition characterized by alternating asymptomatic and symptomatic states [6,7]. Moreover, considering that parasite control did not prevent VL relapse in the R group, it is reasonable to propose that other factors could contribute to the maintenance of high levels of activation in this group.

Persistent activation in HIV infection as well as in VL/HIV co-infection has been associated with microbial translocation from the intestinal lumen into the bloodstream [24,27,28,40,41]. In the current study, VL/HIV-R patients showed higher levels of sCD14, which were positively correlated with the elevated levels of plasma LPS, suggesting that sCD14 is biologically active. The activation of monocytes/macrophages via sCD14 levels contributed not only to the heightened degree of systemic activation through cytokine release but also to the impairment of immune reconstitution because sCD14 levels were negatively correlated with CD4^+^ T cell counts. These data indicate that microbial translocation is an additional factor contributing to frequent VL relapses in patients with HIV infection because only VL/HIV-R patients maintained persistent high levels of sCD14 and LPS after viral and parasite control was achieved. Considering that the sCD14 levels were positively correlated with the parasite load [data not shown], further studies will be necessary to elucidate whether *L*. *infantum* infection can also affect the gut-associated lymphoid tissue (GALT) [42,43]. It is currently believed that the role of *L*. *infantum* is likely indirect because, similar to what has been described for HIV [24,44], this parasite can also be involved in some damage to the gut-associated lymphoid tissue (GALT) [42–43]. Thus, both pathogens can contribute to increased intestinal permeability and consequent microbial translocation, which in turn can maintain the activation status in co-infected patients. Early results from our group showed that LPS levels were augmented in VL patients only during the active phase of disease [25].

In this scenario, the clinical condition of VL/HIV patients can be aggravated by the consequences of immune activation, referred to as accelerated aging of the immune system. Herein, it was hypothesized that the increased levels of chronic activation in VL/HIV co-infection could play a crucial role in promoting the rapid decline of immune system competence, making immunosenescence an additional factor that contributes to the recurrence of VL in HIV-infected patients. In fact, co-infected patients had high percentages of senescent CD4^+^ and CD8^+^ T lymphocytes, which reflects chronic immune activation that does not change despite the use of HAART and maintenance of anti-*Leishmania* treatment. This result is consistent with what has been observed in VL/HIV patients from the Mediterranean basin [30]. Our data confirm this finding through a prospective study design and in patients presenting two distinct clinical outcomes: relapse or no relapse. However, the degree of senescence did not differ between the VL/HIV-R and VL/HIV-NR groups, suggesting that the quantitative accumulation of terminally differentiated cells is not the only important factor. Indeed, immunosenescence includes a lower capacity to respond to new antigens and an exhaustion of primary resources, which can lead to loss of viral load control and progression towards HIV disease [34,35]. Considering that the CD4^+^ T cell count was negatively correlated with cellular activation and that activation is the primary cause of immunosenescence [data not shown], there are two possible explanations for the deficient T cell reconstitution: 1) a deficit in T lymphocyte function due to the impairment of proliferative capacity and cytokine production following parasite or viral antigen-related stimulation and 2) decreased thymic output or a failure to mobilize peripheral T cell compartments. Because the absence of absolute recovery of CD4^+^ T cells after primary VL is an important predictor of relapse in patients infected with HIV [2], ongoing studies in this cohort of patients will address these possibilities.

In addition to the involvement of T lymphocytes in co-infection pathogenesis, a high degree of B cell activation may be inferred because elevated levels of anti-*Leishmania* IgG were observed, particularly in VL/HIV-R patients. Interestingly, the IgG3 levels decreased among NR patients after VL treatment and remained decreased until the end of follow-up, very similar to what was observed for the CD4^+^ and CD8^+^ T lymphocyte activation levels. This result corroborates what has been described in the tegumentary form of leishmaniasis [39], suggesting IgG3 as a possible clinical remission marker for VL that deserves more attention.

In conclusion, our main finding is the observation that HIV-infected patients with recurrent VL have a different immunological profile compared to VL/HIV patients with only one lifelong VL episode, even when they receive the same medications for viral load control and anti-*Leishmania* prophylactic therapy. Although the activation levels were significantly different between the R and NR groups, it was not possible to demonstrate that the differences in the clinical outcomes arise from the process of immunological aging, which may have been due to an insufficiently long follow-up period or the influence of other markers not included in this analysis. Additionally, the quality of the specific effector immune response may be directly related to the different clinical behaviors observed in this study.

Finally, chronically activated immune systems were observed in the patients, even when under secondary anti-*Leishmania* prophylaxis, suggesting that other factors may be associated with the maintenance of an inefficient, hyperactive immune state, including microbial translocation or T cell compartment exhaustion. These findings help to improve our understanding of the mechanisms underlying relapses and reinforce how important early diagnosis and treatment are in reducing immune suppression. Future studies will be important to demonstrate the applicability of these results in the clinical management of these patients in terms of predicting their clinical prognosis as well as deciding whether to prescribe secondary prophylaxis.

## Supporting Information

S1 Fig Gating strategy for evaluating the degree of cellular activation of T lymphocytes in visceral leishmaniasis/HIV (VL/HIV)-co-infected patients. The population of CD3^+^ T lymphocytes (region 2) in the region bounded as total lymphocytes (region 1) was defined. Then, the respective lymphocyte subpopulations, namely, CD4^+^ T and CD8^+^ T cells (regions 3 and 4, respectively) in the CD3^+^ T cell gate were defined. Finally, the coexpression of the HLA-DR and CD38 molecules on CD4^±^ and CD8^±^ T cells was determined from an analysis of the dot plots. The figure shows a representative profile of a non-relapsing patient with VL/HIV.
(TIF)

S2 Fig Gating strategy for evaluating the degree of cellular senescence of T lymphocytes in visceral leishmaniasis/HIV (VL/HIV)-co-infected patients. The population of CD3^±^ T lymphocytes (region 2) in the region bounded as total lymphocytes (region 1) was defined. Then, the respective lymphocyte subpopulations, namely, CD4^+^ T and CD8^+^ T cells (regions 3 and 4, respectively) in the CD3^+^ T cell gate were defined. Finally, the coexpression of the CD57 and CD27 molecules on CD4^±^ and CD8^±^ T cells was determined from an analysis of the dot plots. The figure shows a representative profile of a non-relapsing patient with VL/HIV.
(TIF)

S3 Fig Titers of anti-*Leishmania infantum* immunoglobulin G (IgG) and the IgG1 isotype in visceral leishmaniasis/HIV (VL/HIV)-co-infected patients. IgG and IgG1 levels in the relapsing (R) and non-relapsing (NR) groups during the entire follow-up. The red dashed line represents the median values of the IgG and IgG1 levels in healthy controls (medians: 0.85 and 0.19; interquartile ranges: 0.5–1.1 and 0.08–0.55, respectively). Each symbol represents one patient, and the color refers to the same patient at different stages of follow up. The horizontal bars represent the median values. 6 mpt: 6 months post-treatment; 12 mpt: 12 months post-treatment.
(TIF)

S1 Table Copies per mL numbers for viral RNA and kDNA of *Leishmania (L*.*) infantum* presented by visceral leishmaniasis/HIV (VL/HIV) co-infected patients. (DOC)

## Abbreviations

HAART: highly active antiretroviral therapy
VL: visceral leishmaniasis

## References

[1] Lindoso JA, Cota GF, da Cruz AM, Goto H, Maia-Elkhoury ANS, Romero GAS, et al Visceral Leishmaniasis and HIV Coinfection in Latin America. PLoS Negl Trop Dis. 2014;8(9):e3136 10.1371/journal.pntd.0003136 25233461 PMC4169383

[2] Cota GF, de Sousa MR, Rabello A, et al Predictors of visceral leishmaniasis relapse in hiv-infected patients: A systematic review. PLoS Negl Trop Dis. 2011;5(6):1–8. 10.1371/journal.pntd.0001153 PMC3110161 21666786

[3] Cota GF, de Sousa MR, Fereguetti TO, Rabello A. Efficacy of Anti-Leishmania Therapy in Visceral Leishmaniasis among HIV Infected Patients: A Systematic Review with Indirect Comparison. PLoS Negl Trop Dis. 2013;7(5):e1153. 10.1371/journal.pntd.0002195 PMC3642227 23658850

[4] Cota GF, de Sousa MR, de Freitas Nogueira BM, Gomes LI, Oliveira E, Assis TSM, et al Comparison of Parasitological, Serological, and Molecular Tests for Visceral Leishmaniasis in HIV-Infected Patients: A Cross-Sectional Delayed-Type Study. Am J Trop Med Hyg. 2013;89(3):570–7. 10.4269/ajtmh.13-0239 23836568 PMC3771302

[5] De la Rosa R, Pineda J a, Delgado J, Macias J, Morillas F, Martin-Sanchez J, et al Influence of highly active antiretroviral therapy on the outcome of subclinical visceral leishmaniasis in human immunodeficiency virus-infected patients. Clin Infect Dis. 2001;32(4):633–5. 10.1086/318708 11181128

[6] Del Giudice P, Mary-Krause M, Pradier C, Grabar S, Dellamonica P, Marty P, et al Impact of highly active antiretroviral therapy on the incidence of visceral leishmaniasis in a French cohort of patients infected with human immunodeficiency virus. J Infect Dis. 2002;186(9):1366–70. 10.1086/344325 12402211

[7] Cruz I, Nieto J, Moreno J, Canavate C, Desjeux P, Alvar J. *Leishmania*/HIV co-infections in the second decade. Indian J Med Res.; 2006;123(3):357–88. 16778317

[8] Alvar J, Aparicio P, Aseffa a., Den Boer M, Canavate C, Dedet J-P, et al The Relationship between Leishmaniasis and AIDS: the Second 10 Years. Clin Microbiol Rev. 2008;21(2):334–59. 10.1128/CMR.00061-07 18400800 PMC2292576

[9] Okwor I, Uzonna JE. The immunology of *Leishmania*/HIV co-infection. Immunol Res. 2013;56(1):163–71. 10.1007/s12026-013-8389-8 23504228

[10] Monge-Maillo B, Norman FF, Cruz I, Alvar J, López-Vélez R. Visceral Leishmaniasis and HIV Coinfection in the Mediterranean Region. PLoS Negl Trop Dis. 2014;8(8):e3021 10.1371/journal.pntd.0003021 25144380 PMC4140663

[11] Demarchi IG, Silveira TG V, Ferreira ICP, Lonardoni MVC. Effect of HIV protease inhibitors on New World *Leishmania*. Parasitol Int.; 2012;61: 538–44. 10.1016/j.parint.2012.04.006 22579524

[12] Santos LO, Vitorio BS, Branquinha MH, Pedroso e Silva CM, Santos a. LS, d’Avila-Levy CM. Nelfinavir is effective in inhibiting the multiplication and aspartic peptidase activity of *Leishmania* species, including strains obtained from HIV-positive patients. J Antimicrob Chemother. 2013;68: 348–353. 10.1093/jac/dks410 23109184 PMC3543121

[13] Cota GF, de Sousa MR, de Mendonça ALP, Patrocinio A, Assunção LS, de Faria SR, et al *Leishmania*-HIV Co-infection: Clinical Presentation and Outcomes in an Urban Area in Brazil. PLoS Negl Trop Dis. 2014;8(4):2–8. 10.1371/journal.pntd.0002816 PMC3990491 24743472

[14] Alemayehu M, Wubshet M, Mesfin N. Magnitude of visceral leishmaniasis and poor treatment outcome among HIV patients: metaanalysis and systematic review. HIV/AIDS—Res Palliat Care. 2016;8: 75–81. 10.2147/HIV.S96883 PMC4809333 27042142

[15] Casado JL, Lopez-Velez R, Pintado V, Quereda C, Antela A, Moreno S. Relapsing visceral leishmaniasis in HIV-infected patients undergoing successful protease inhibitor therapy. Eur J Clin Microbiol Infect Dis.; 2001;20(3):202–5. 11347673 10.1007/s100960100457

[16] Alexandrino-de-Oliveira P, Santos-Oliveira JR, Dorval MEC, da Costa FCB, Pereira GROL, da Cunha R V, et al HIV/AIDS-associated visceral leishmaniasis in patients from an endemic area in Central-west Brazil. Mem Inst Oswaldo Cruz. 2010;105(5):692–7. 20835619 10.1590/s0074-02762010000500016

[17] Villanueva JL, Alarcón a, Bernabeu-Wittel M, Cordero E, Prados D, Regordán C, et al Prospective evaluation and follow-up of European patients with visceral leishmaniasis and HIV coinfection in the era of highly active antiretroviral therapy. Eur J Clin Microbiol Infect Dis. 2000;19(10):798–801. 11117648 10.1007/s100960000364

[18] Santos-Oliveira JR, Giacoia-Gripp CBW, Alexandrino de Oliveira P, Amato VS, Lindoso JÂL, Goto H, et al High levels of T lymphocyte activation in *Leishmania*-HIV co-infected individuals despite low HIV viral load. BMC Infect Dis. 2010;10(1):358. 21171992 10.1186/1471-2334-10-358 PMC3022832

[19] Da-Cruz AM, Mattos M, Oliveira-Neto MP, Coutinho Z, Machado ES, Coutinho SG. Cellular immune responses to *Leishmania braziliensis* in patients with AIDS-associated American cutaneous leishmaniasis. Trans R Soc Trop Med Hyg. 2000;94(5):569–71. 11132391 10.1016/s0035-9203(00)90090-7

[20] Santos-Oliveira JR, Da-Cruz AM, Pires LHS, Cupolillo E, Kuhls K, Giacoia-Gripp CBW, et al Case report: Atypical lesions as a sign of cutaneous dissemination of visceral leishmaniasis in a human immunodeficiency virus-positive patient simultaneously infected by two viscerotropic *Leishmania* species. Am J Trop Med Hyg. 2011;85(1):55–9. 21734124 10.4269/ajtmh.2011.10-0398 PMC3122343

[21] Zijlstra EE. PKDL and Other Dermal Lesions in HIV Co-infected Patients with Leishmaniasis: Review of Clinical Presentation in Relation to Immune Responses. PLoS Negl Trop Dis. 2014;8(11):e3258 10.1371/journal.pntd.0003258 25412435 PMC4238984

[22] Gómez Senent S, Adan Merino L, Mora Sanz P. Kala azar con afectación gástrica. Gastroenterol Hepatol. 2009;32(3):176–7. 10.1016/j.gastrohep.2008.09.021 19233514

[23] Luz KG, Tuon FF, Irma M, Duarte S, Maia GM, Matos P, et al Cytokine expression in the duodenal mucosa of patients with visceral leishmaniasis. Rev Soc Bras Med Trop. 2010;43(4):393–5. 20802937 10.1590/s0037-86822010000400011

[24] Brenchley JM, Price D a, Schacker TW, Asher TE, Silvestri G, Rao S, et al Microbial translocation is a cause of systemic immune activation in chronic HIV infection. Nat Med. 2006;12(12):1365–71. 10.1038/nm1511 17115046

[25] Santos-Oliveira JR, Regis EG, Leal CRB, Cunha R V., Bozza PT, Da-Cruz AM. Evidence that lipopolisaccharide may contribute to the cytokine storm and cellular activation in patients with visceral leishmaniasis. PLoS Negl Trop Dis. 2011;5(7):e1198 10.1371/journal.pntd.0001198 21765960 PMC3134430

[26] Peruhype-Magalhães V, Martins-Filho OA., Prata a., Silva LD A., Rabello A., Teixeira-Carvalho A., et al Mixed inflammatory/regulatory cytokine profile marked by simultaneous raise of interferon-ɣ and interleukin-10 and low frequency of tumour necrosis factor-α^+^ monocytes are hallmarks of active human visceral Leishmaniasis due to *Leishmania* chag. Clin Exp Immunol. 2006;146(1):124–32. 10.1111/j.1365-2249.2006.03171.x 16968407 PMC1809731

[27] Klatt NR, Funderburg NT, Brenchley JM. Microbial translocation, immune activation, and HIV disease. Trends Microbiol. 2013;21(1):6–13. 10.1016/j.tim.2012.09.001 23062765 PMC3534808

[28] Santos-Oliveira JR, Regis EG, Giacoia-Gripp CBW, Valverde JG, Alexandrino-De-Oliveira P, Lindoso JÂL, et al Microbial translocation induces an intense proinflammatory response in patients with visceral leishmaniasis and HIV type 1 coinfection. J Infect Dis. 2013;208:57–66. 10.1093/infdis/jit135 23539743

[29] Medrano FJ, Rey C, Leal M, Cañavate C, Rubio a., Sánchez-Quijano a., et al Dynamics of serum cytokines in patients with visceral leishmaniasis and HIV co-infection. Clin Exp Immunol. 1998;114(3):403–7. 10.1046/j.1365-2249.1998.00733.x 9844050 PMC1905121

[30] Casado JL, Abad-Fernández M, Moreno S, Pérez-Elías MJ, Moreno a, Bernardino JI, et al Visceral leishmaniasis as an independent cause of high immune activation, T-cell senescence, and lack of immune recovery in virologically suppressed HIV-coinfected patients. HIV Med. 2015;16(4):240–8. 10.1111/hiv.12206 25604328

[31] Chou JP, Ramirez CM, Wu JE, Effros RB. Accelerated Aging in HIV/AIDS: Novel Biomarkers of Senescent Human CD8+ T Cells. PLoS One. 2013;8(5):1–7. 10.1371/journal.pone.0064702 PMC3661524 23717651

[32] Kaushal H, Bras-Gonçalves R, Negi NS, Lemesre J-L, Papierok G, Salotra P. Role of CD8+ T cells in protection against *Leishmania donovani* infection in healed Visceral Leishmaniasis individuals. BMC Infect Dis. 2014;14:653 10.1186/s12879-014-0653-6 25471494 PMC4258298

[33] Serrano-Villar S, Pérez-Elías MJ, Dronda F, Casado JL, Moreno A, Royuela A, et al Increased risk of serious non-AIDS-related events in HIV-infected subjects on antiretroviral therapy associated with a low CD4/CD8 ratio. PLoS One. 2014;9(1):e85798 10.1371/journal.pone.0085798 24497929 PMC3907380

[34] Appay V, Sauce D. Immune activation and inflammation in HIV infection: causes and consequences. J Pathol. 2008;214(2):231–41. 10.1002/path.2276 18161758

[35] Deeks SG, Verdin E, McCune JM. Immunosenescence and HIV. Curr Opin Immunol. 2012;24(4):501–6. 10.1016/j.coi.2012.05.004 22658763

[36] Wortmann G, Sweeney C, Houng HS, Aronson N, Stiteler J, Jackson J, et al Rapid diagnosis of Leishmaniasis by fluorogenic polymerase chain reaction. Am J Trop Med Hyg. 2001;65(5):583–7. 11716118 10.4269/ajtmh.2001.65.583

[37] Gomes LI, Gonzaga FM, Morais-Teixeira De E, de Souza-Lima BS, Freire V V., Rabello A. Validation of quantitative real-time PCR for the in vitro assessment of antileishmanial drug activity. Exp Parasitol. 2012;131(2):175–9. 10.1016/j.exppara.2012.03.021 22475774

[38] Overbergh L, Valckx D, Waer M, Mathieu C. Quantification of murine cytokine mRNAs using real time quantitative reverse transcriptase PCR. Cytokine. 1999;11(4):305–12. 10.1006/cyto.1998.0426 10328870

[39] Fagundes-Silva GA, Vieira-Goncalves R, Nepomuceno MP, de Souza MA, Favoreto S, Oliveira-Neto MP, et al Decrease in anti-*Leishmania* IgG3 and IgG1 after cutaneous leishmaniasis lesion healing is correlated with the time of clinical cure. Parasite Immunol. 2012;34(10):486–91. 10.1111/j.1365-3024.2012.01379.x 22742527

[40] Brenchley JM, Schacker TW, Ruff LE, Price D a, Taylor JH, Beilman GJ, et al CD4+ T cell depletion during all stages of HIV disease occurs predominantly in the gastrointestinal tract. J Exp Med. 2004;200(6):749–59. 10.1084/jem.20040874 15365096 PMC2211962

[41] Mehandru S, Poles MA, Tenner-Racz K, Horowitz A, Hurley A, Hogan C, et al Primary HIV infection is associated with preferential depletion of CD4+ T lymphocytes from effector sites in the gastrointestinal tract. J Exp Med. 2004;200(6):761–70. 10.1084/jem.20041196 15365095 PMC2211967

[42] Hicks L, Kant P, Tay PH, Vincini V, Schuster H, Rotimi O, et al Visceral Leishmaniasis presenting with intestinal failure: a case report and literature review. Eur J Gastroenterol Hepatol. 2009;21(1):117–22. 10.1097/MEG.0b013e32830e6fdb 19011572

[43] Baba CS, Makharia GK, Mathur P, Ray R, Gupta SD, Samantaray JC. Chronic diarrhea and malabsorption caused by *Leishmania donovani*. Indian J Gastroenterol; 2006;25(6):309–10. 17264434

[44] Marchetti G, Tincati C, Silvestri G. Microbial translocation in the pathogenesis of HIV infection and AIDS. Clin Microbiol Rev. 2013;26(1):2–18 10.1128/CMR.00050-12 23297256 PMC3553668

