# Supplementary figures and images for: Immune Activation and Bacterial Translocation: A Link between Impaired Immune Recovery and Frequent Visceral Leishmaniasis Relapses in HIV-Infected Patients
Source: PLoS One. 2016 Dec 1;11(12):e0167512. doi: 10.1371/journal.pone.0167512 (PMC5132299; doi:10.1371/journal.pone.0167512)

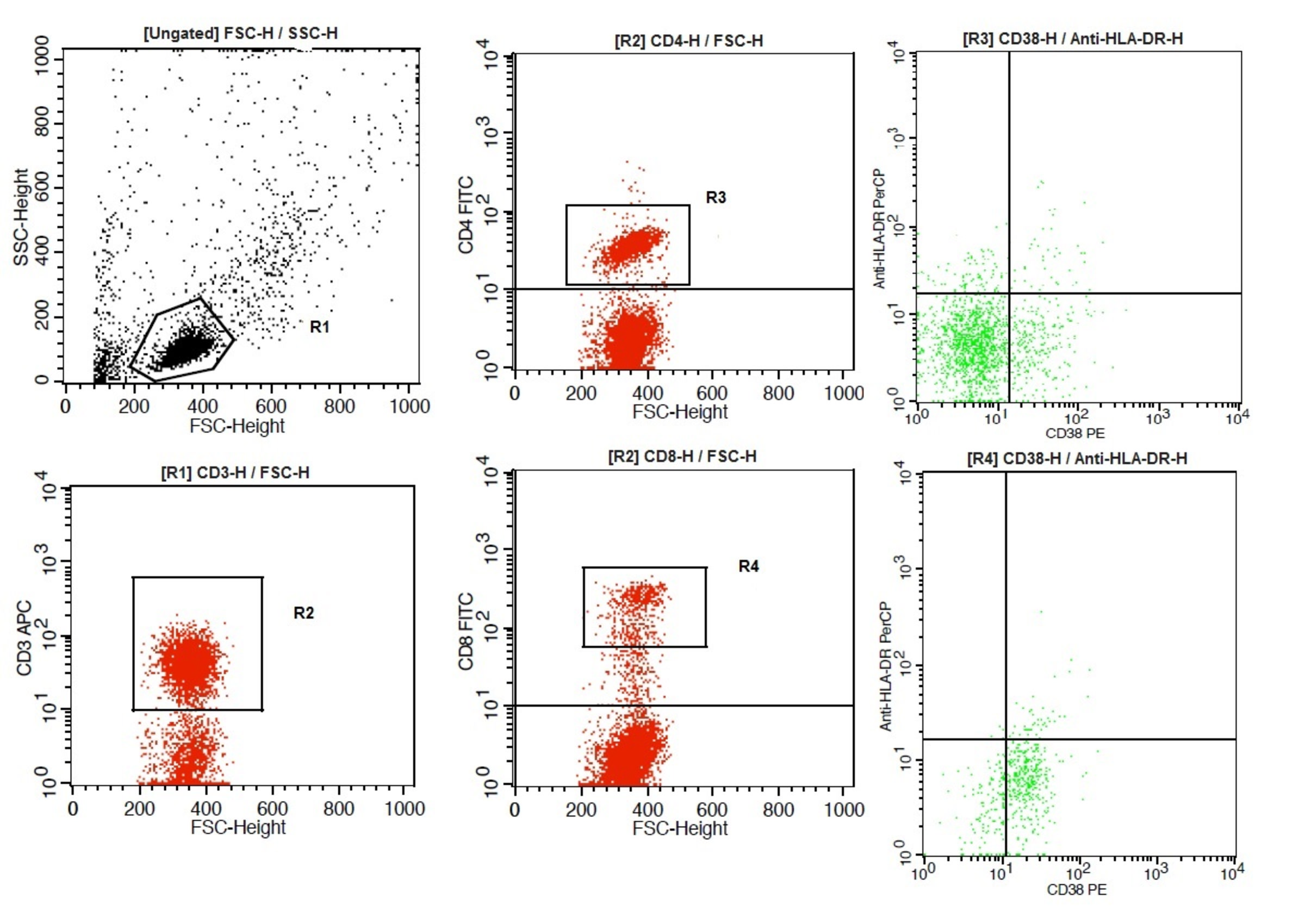

Supplement: S1 Fig — The population of CD3+ T lymphocytes (region 2) in the region bounded as total lymphocytes (region 1) was defined. Then, the respective lymphocyte subpopulations, namely, CD4+ T and CD8+ T cells (regions 3 and 4, respectively) in the CD3+ T cell gate were defined. Finally, the coexpression of the HLA-DR and CD38 molecules on CD4± and CD8± T cells was determined from an analysis of the dot plots. The figure shows a representative profile of a non-relapsing patient with VL/HIV. (TIF) [file pone.0167512.s001.tif]

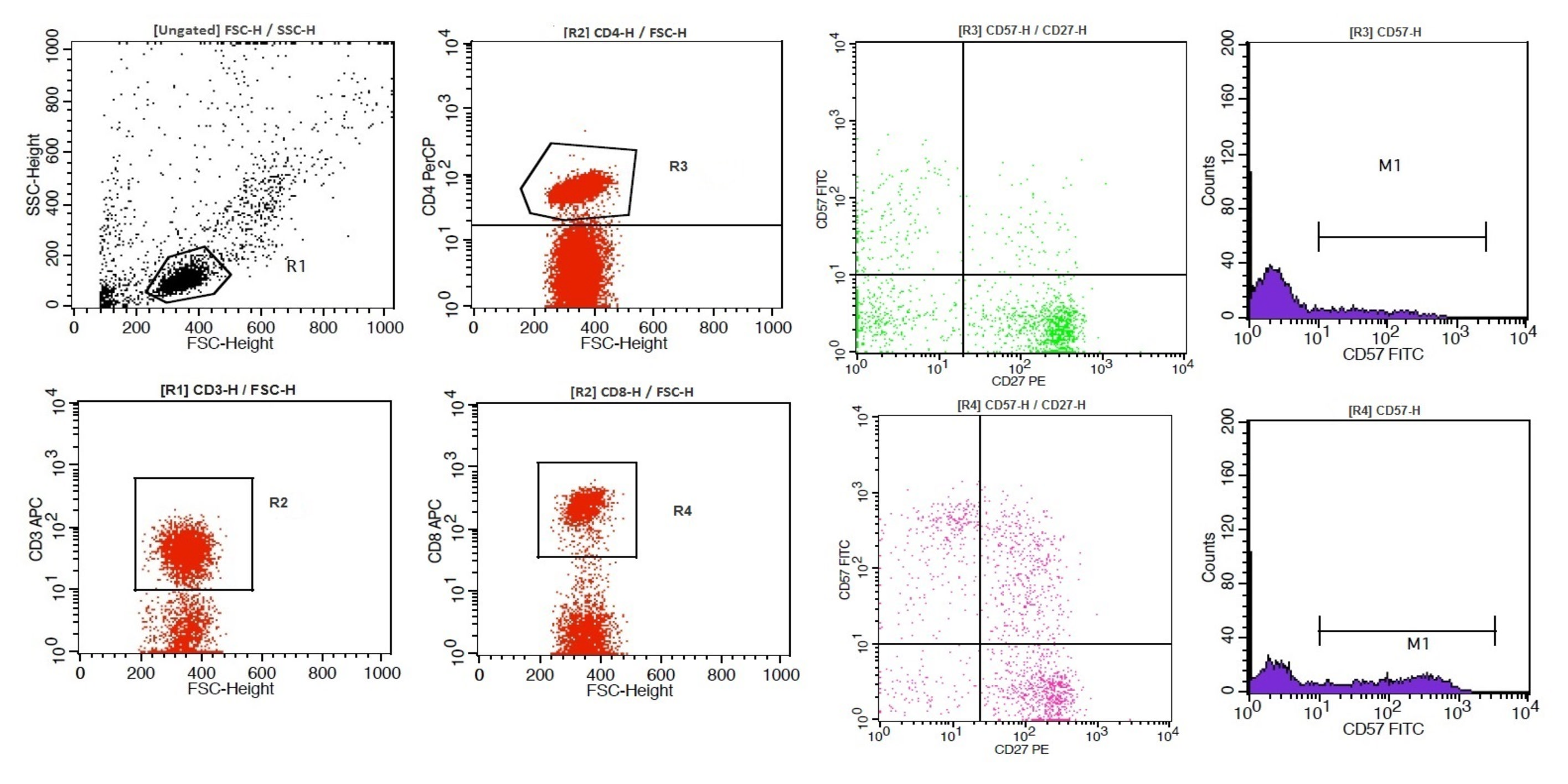

Supplement: S2 Fig — The population of CD3± T lymphocytes (region 2) in the region bounded as total lymphocytes (region 1) was defined. Then, the respective lymphocyte subpopulations, namely, CD4+ T and CD8+ T cells (regions 3 and 4, respectively) in the CD3+ T cell gate were defined. Finally, the coexpression of the CD57 and CD27 molecules on CD4± and CD8± T cells was determined from an analysis of the dot plots. The figure shows a representative profile of a non-relapsing patient with VL/HIV. (TIF) [file pone.0167512.s002.tif]

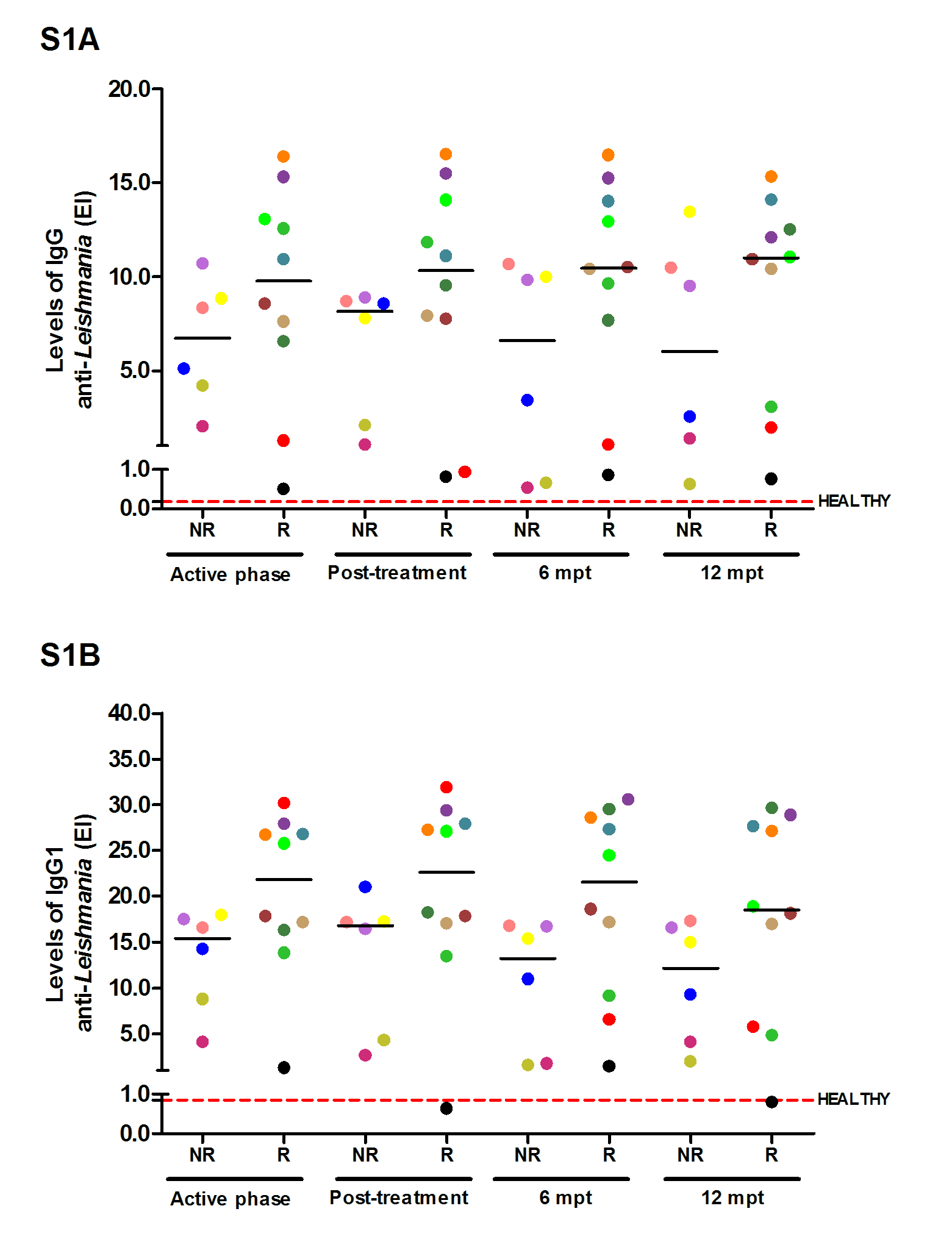

Supplement: S3 Fig — IgG and IgG1 levels in the relapsing (R) and non-relapsing (NR) groups during the entire follow-up. The red dashed line represents the median values of the IgG and IgG1 levels in healthy controls (medians: 0.85 and 0.19; interquartile ranges: 0.5–1.1 and 0.08–0.55, respectively). Each symbol represents one patient, and the color refers to the same patient at different stages of follow up. The horizontal bars represent the median values. 6 mpt: 6 months post-treatment; 12 mpt: 12 months post-treatment. (TIF) [file pone.0167512.s003.tif]
