# Supplementary material for: Immune Activation and Bacterial Translocation: A Link between Impaired Immune Recovery and Frequent Visceral Leishmaniasis Relapses in HIV-Infected Patients
Source: PLoS One. 2016 Dec 1;11(12):e0167512. doi: 10.1371/journal.pone.0167512 (PMC5132299; doi:10.1371/journal.pone.0167512)
Supplement: S1 Table — (DOC) [file pone.0167512.s004.doc]

***S1 Table: Copies per mL numbers for viral RNA and kDNA of Leishmania (L.) infantum presented by visceral leishmaniasis/HIV (VL/HIV) co-infected patients.***

|  |  | **Active Phase** | | **Post-treatment** | | **6 months post-treatment (mpt)** | | **12 months post-treatment (mpt)** | |
| --- | --- | --- | --- | --- | --- | --- | --- | --- | --- |
|  |  | **Viral load (RNA copies/mL)** | **Parasite load (kDNA copies/mL)** | **Viral load (RNA copies/mL)** | **Parasite load (kDNA copies/mL)** | **Viral load (RNA copies/mL)** | **Parasite load (kDNA copies/mL)** | **Viral load (RNA copies/mL)** | **Parasite load (kDNA copies/mL)** |
| **HLV01** | **NON**  **RELAPSE** | **20272** | **38171** | **571** | **Undet.** | **Undet.** | **Undet.** | **Undet.** | **1.3** |
| **HLV06** |  | **55** | **206162** | **Undet.** | **Undet.** | **Undet.** | **Undet.** | **Undet.** | **Undet.** |
| **HLV07** |  | **Undetectable** | **159550** | **Undetectable** | **Undetectable** | **Undetectable** | **Undetectable** | **Undetectable** | **Undetectable** |
| **HLV013** |  | **153316** | **Undetectable** | **1118** | **Undetectable** | **Undetectable** | **Undetectable** | **60 (10 mpt)** | **Undetectable** |
| **HLV016** |  | **Undetectable** | **78** | **Undetectable** | **Undetectable** | **Undetectable** | **Undetectable** | **Undetectable** | **Undetectable** |
| **HLV025** |  | **51** | **506308** | **Undetectable** | **Undetectable** | **Undetectable** | **Undetectable** | **1061258** | **Undetectable** |
| **HLV03** | **RELAPSE** | **Undetectable** | **46989** | **Undetectable** | **Undetectable** | **Abandoned follow-up** | | | |
| **HLV05** |  | **1146** | **37271** | **181** | **Undetectable** | **394230** | **Undetectable** | **157833** | **Undetectable** |
| **HLV09** |  | **Undetectable** | **41.1** | **Undetectable** | **12718** | **Undetectable** | **0.8** | **Undetectable** | **1334** |
| **HLV010** |  | **Undetectable** | **3147** | **--** | **1467** | **Undetectable** | **Undetectable** | **--** | **Undetectable** |
| **HLV012** |  | **405** | **0.2** | **194** | **3024000** | **23004** | **5400** | **Death** | |
| **HLV017** |  | **Undetectable** | **115454** | **Undetectable** | **430** | **Undetectable** | **2663** | **Undetectable** | **--** |
| **HLV019** |  | **Undetectable** | **40177** | **Undetectable** | **Undetectable** | **Undetectable** | **Undetectable** | **--** | **23.5** |
| **HLV021** |  | **56543** | **813** | **4897** | **622** | **86899** | **3262** | **178326** | **Undetectable** |
| **HLV022** |  | **Undetectable** | **38281** | **60** | **220** | **--** | **7909** | **82** | **54** |
| **HLV023** |  | **47** | **25118** | **Undetectable** | **6369** | **339** | **2030** | **--** | **1.1** |
| **HLV024** |  | **135957** | **81656** | **68075** | **Undetectable** | **--** | **24705** | **190839** | **4442** |
| **HLV026** |  | **250310** | **15701** | **7311** | **116** | **133219** | **Undetectable** | **--** | **Undetectable** |
